# Monitoring Cardiac Function Using Seismocardiography Post-Transcatheter Aortic Valve Implantation

**DOI:** 10.1016/j.jacadv.2025.101664

**Published:** 2025-03-14

**Authors:** Jyotpal Singh, Maria Gagarinova, Chase J. Ellingson, Jeffrey Booker, Philippe Pibarot, J. Patrick Neary, Payam Dehghani

**Affiliations:** a Prairie Vascular Research Inc, Regina, Saskatchewan, Canada; b Department of Kinesiology and Health Studies, University of Regina, Regina, Saskatchewan, Canada; c Quebec Heart & Lung Institute–Laval University, Quebec City, Quebec, Canada

**Keywords:** aortic valve stenosis, seismocardiography, Tei index, transcatheter aortic valve implantation

Transcatheter aortic valve implantation (TAVI) can decrease left ventricular ejection time (LVET)^1^ and increase the Tei index^1^ and markers of diastolic function, such as isovolumic relaxation time (IVRT) and E-wave deceleration time.^2^

**What is the clinical question being addressed?**

What are the changes in seismocardiogram-derived cardiac intervals in patients undergoing transcatheter aortic valve implantation?

**What is the main finding?**

The SCG-derived LVET decreased, and IVRT increased 24 hours post-TAVI, increasing the SCG-derived Tei index.

Echocardiography is the most common assessment of hemodynamics and cardiac function. Seismocardiography (SCG) is less resource-intensive and monitors hemodynamic function by analyzing the vibrations produced by the heartbeat. This allows for identifying the opening and closing of the heart valves.^3^ This study aimed to characterize SCG-derived changes in hemodynamic function following TAVI.

## Methods

The local research ethics board approved this study, and all participants signed an informed consent form before enrolling. Patients with symptomatic (NYHA functional class ≥2) aortic stenosis (AS) scheduled for nonemergent TAVI were enrolled prospectively.

An SCG sensor (LLA Recordis, LLA Technologies) was placed directly on the patient’s chest, 1 cm above the xiphoid process. The patient rested supine for ∼1 minute before each 1-minute SCG measurement: the morning of the TAVI and 24 hours following TAVI. An in-house, independent (proprietary) algorithm (LLA Technologies Inc) extracted the cardiac features from the heart vibrations.^3^ In a supine position, the x-axis pointed laterally from left to right, the y-axis pointed from head to foot, and the z-axis pointed from back to front. The LLA Recordis waveform and identification of these fiducial points are documented in their patent.^4^ An in-house algorithm identified points of aortic valve opening (AVO), aortic valve closure (AVC), mitral valve opening (MVO), and mitral valve closure (MVC). Temporal intervals of ejection (AVO−AVC), diastole (MVC−MVO timing), isovolumic contraction time (IVCT) (MVC−AVO), and IVRT (AVC−MVO) were calculated.^3^ The force produced by the left ventricle (LV) (twist force) and left atrium (LA) (atrial systole) contraction, assessed by the magnitude of the vibration for each heartbeat, was identified by the cardiac sensor in milligravity (mG). The SCG-derived Tei index ([IVRT + IVCT]/ejection time; also known as myocardial performance index^3^), systolic performance index (SPI) ([IVCT/ejection time]), and diastolic performance index (DPI; [IVRT/ejection time]) were calculated.

A paired sample t-test was used to compare the pre-TAVI SCG data to approximately 24 hours post-TAVI. The cardiac cycle data are presented as mean ± SD, with the change from pre to 24 hours post calculated as the difference in mean ± combined SD. The SCG-derived contractile parameters and echocardiography data are presented as median (IQR). Data were analyzed using R version 4.1.2 (R Foundation for Statistical Computing).

## Results

Of 100 patients (age 80 ± 6 years; n = 69 male), 85 had hypertension, 68 had dyslipidemia, and 38 had diabetes. The majority of the patients were on a statin (n = 69), antiplatelet (n = 67), and/or diuretic (n = 56). On echocardiography before TAVI, participants had a left ventricular ejection fraction of 57% (48, 61%), mean transvalvular gradient of 40 mm Hg (32, 45 mm Hg), and aortic valve area of 0.8 cm^2^ (0.7, 1.0 cm^2^). Echocardiography performed within 24 hours post-TAVI showed a left ventricular ejection fraction of 58% (50, 63%), mean transvalvular gradient of 8 mm Hg (6, 11 mm Hg), and aortic valve area of 2.6 cm^2^ (2.1, 3.1 cm^2^).

Pre- to post-TAVI, there was an increase in heart rate from 71 to 75 bpm (*P* < 0.01). SCG-derived LVET decreased from 333 ms to 297 ms (*P* < 0.001), and SCG-derived IVRT increased from 85 ms to 90 ms (*P* < 0.001). SCG-derived Tei index increased from 0.37 to 0.43 (*P* < 0.001), while the SPI increased from 0.11 to 0.12 (*P* < 0.01), and DPI increased from 0.26 to 0.31 (*P* < 0.001). There was an increase in twist force from 9 (6, 11) to 11 (7, 14) mG (*P* < 0.001) and an increase in atrial systole from 5 (4, 7) to 8 (6, 11) mG (*P* < 0.001) at the z-axis, and from 3 (2, 4) to 4 (3, 6) mG (*P* < 0.01) at the x-axis (Table 1).

**Table 1:** Seismocardiogram-Derived Cardiac Function Parameters

	Pre-TAVI	24 H Post-TAVI	Pre- to 24-H Post-Change	Significance
Heart rate (beats/min)	71 ± 12	75 ± 10	4 ± 16	<0.01
Ejection time (ms)	333 ± 54	297 ± 45	36 ± 70	<0.001
Diastolic time (ms)	418 ± 89	402 ± 83	16 ± 122	0.08
IVRT (ms)	85 ± 10	90 ± 12	5 ± 16	<0.001
IVCT (ms)	34 ± 6	34 ± 6	0 ± 8	0.89
SPI	0.11 ± 0.02	0.12 ± 0.03	0.01 ± 0.04	<0.01
DPI	0.26 ± 0.05	0.31 ± 0.06	0.05 ± 0.08	<0.001
Tei index	0.37 ± 0.07	0.43 ± 0.07	0.06 ± 0.10	<0.001
Twist force (mG)	9 (6-11)	11 (7-14)	2 (−1 to 6)	<0.001
Atrial systole (mG)	5 (4-7)	8 (6-11)	3 (0-6)	<0.001

Values are mean ± SD or median (IQR).

DPI = diastolic performance index; IVCT = isovolumic contraction time; IVRT = isovolumic relaxation time; SPI = systolic performance index; TAVI = transcatheter aortic valve implantation.

## Discussion

This is the first study to assess hemodynamic parameters pre- and post-TAVI using an SCG device. Post-TAVI, there was a decrease in SCG-derived LVET, while SCG-derived Tei index and contractile force increased.

Relieving obstruction in the left ventricular outflow tract reduces LVET,^1^ potentially due to increased blood pressure and stroke volume and reduced systemic vascular resistance. This study showed for the first time that these similar adaptive changes can be illustrated by using SCG. As such, the routine use of SCG can provide continuous feedback to monitor patient progression following TAVI in a rapid, resource-sparing method. In turn, assessment of cardiac function by SCG following TAVI offers an opportunity for immediate triage should there be any notable changes, such as a prolonged LVET.

Previous research suggests that relieving left ventricular outflow tract obstruction increases the Tei index.^1^ Similarly, we showed that the SCG-derived Tei index increased post-TAVI. The SPI and DPI values, pre- and post-TAVI, have not previously been reported in patients with AS. Given their inverse correlation to ejection time, our findings indicate that SCG-derived SPI and DPI values postintervention resemble those seen in a healthy population.^3^

Diastolic function is also affected following TAVI. Previous echocardiography-based studies have shown that the E/A and E/e ratios decrease while IVRT and E-wave deceleration time increase.^2^ The increase in IVRT post-TAVI reflects a decrease in LA pressure.^2^ We showed that the SCG-derived IVRT increased post-TAVI, which agrees with previous literature using echocardiography.^2^ As such, IVRT can be a promising marker for assessing restrictive filling and diastolic function following TAVI.

Contractile properties of both the LV and LA have been shown to change post-TAVI. Global longitudinal strain measures the deformation of the LV using strain echocardiography, and global longitudinal strain increases by approximately 3% post-TAVI.^5^ Intriguingly, the twist force measured by SCG in our study, considered a surrogate of contractility,^3^ also increased by 3 mG post-TAVI. Deformation of the LA can also provide insight into atrial contraction. Using strain echocardiography, as the LA fills and stretches, the peak LA longitudinal strain can be measured (peaks in systole at the end of LA filling and before the mitral valve opens).^5^ Coyle et al^5^ also showed that atrial strain increases by approximately 3% post-TAVI,^5^ similar to the 3 mG increase in SCG-derived atrial systole post-TAVI from our analysis.

Broad inclusion criteria, limited SCG-derived hemodynamic variables, and the need for research with simultaneous SCG and echocardiogram correlations in patients with AS limited this study.

## Conclusions

We provide the first study to identify SCG-derived reductions in LVET and increases in the Tei index, IVRT, and contractile forces following TAVI. Using SCG allows continuous observation of cardiac timing and contractility and monitoring patients’ recovery following TAVI without increasing resource utilization.

## Funding support and author disclosures

Dr Neary is a co-author on a patent related to the seismocardiography device used in this study. All other authors have reported that they have no relationships relevant to the contents of this paper to disclose.
